# Hierarchically Graphitic Carbon Structure Derived from Metal Ions Impregnated Harmful Inedible Seaweed as Energy-Related Material

**DOI:** 10.3390/ma17184643

**Published:** 2024-09-21

**Authors:** Yun-Mi Song, Hui Gyeong Park, Jung-Soo Lee

**Affiliations:** Department of Bio-Chemical Engineering, Chosun University, Chosundaegil 146, Dong-gu, Gwangju 61452, Republic of Korea

**Keywords:** porous carbon, graphitic carbon, seaweed, metal catalyst, supercapacitor, EDLC

## Abstract

This study explored the development of hierarchical graphitic carbon structures (HGCs) from harmful inedible seaweed waste harvested in the summer. Elevated sea temperatures during the summer increase the cellulose content of seaweeds, making them unsuitable for consumption. By utilizing seaweed biomass, this study addresses critical marine environmental issues and provides a sustainable solution for promising electrode materials for energy storage devices. The fabrication process involved impregnating seaweed with Ni ions, followed by annealing to create a highly crystalline carbon structure. Subsequent etching produced numerous nano-sized pores and a large surface area (806 m^2^/g), significantly enhancing the number of electrically active sites. The resulting HGCs exhibited a high capacitance and maintained their capacity even after 10,000 cycles in fast-current systems. This innovative approach not only mitigates the environmental burden of seaweed waste but also offers a sustainable method for converting it into efficient energy storage materials.

## 1. Introduction

In recent years, the eco-friendly renewable energy, portable electronics, and electric vehicle markets have focused on high-performance energy storage systems, such as rechargeable batteries and supercapacitors [1,2]. Supercapacitors with distinct advantages such as high power density, long cycle life, stability, and cost-effectiveness will be used in an increasing number of applications as alternative energy storage resources for future rechargeable batteries [3,4,5,6].

A supercapacitor consists of two electrodes: an electrolyte and a separator that electrically isolates the two electrodes. Based on their energy storage mechanism and electrode material, they are divided into pseudocapacitors and electrical double-layer capacitors (EDLCs). Pseudocapacitors store electrical energy by oxidation–reduction reactions occurring at the electrode interface, and EDLCs store electrical energy by the electrostatic accumulation of charge at the electrode/electrolyte interface. EDLCs provide extremely fast energy uptake and transmission, improve power performance, and withstand tens of millions of cycles [7,8,9].

The electrochemical performance of a supercapacitor is highly dependent on the unique properties of the electrode material, such as surface area, electrical conductivity, wetting, and permeability of the electrolyte [10,11,12,13]. Currently, the most commonly used electrode materials are carbon, metal oxides, and conductive polymers. Carbon materials are the most widely used electrode materials because of their wide surface areas, durable inertness, excellent electrical properties, and reasonable costs. Moreover, it is essential to develop low-cost, highly efficient porous carbon materials with high electrical conductivities, increased specific surface areas, and electroactive sites to improve the performance of supercapacitors. Porous carbon produced using metal catalysts has the advantages of small defects, excellent conductivity, controllability up to the nanometer scale, and easy mass production [14,15,16,17,18,19].

Biomass materials are expected to be a valuable resource for the fabrication of porous carbon compared to ever-decreasing fossil resources because of their unique properties, such as renewability, low cost, and environmental friendliness. Thus, several biomass resources have been used as carbon precursors to fabricate porous carbon materials that have shown great potential as supercapacitor electrode materials. Additionally, biomass utilization plays a pivotal role in achieving carbon neutrality. Biomass recycling contributes to carbon neutrality in various ways, including its role in the carbon cycle, replacement of fossil fuels, carbon capture and storage, waste management and resource recycling, and ecosystem protection and restoration, and its nature as a renewable resource. This enables us to simultaneously achieve sustainable energy systems and environmental protection [20,21].

Among various types of biomass, seaweed-based biomass offers numerous advantages for sustainable development and environmental conservation [22]. Harmful and inedible seaweeds pose significant challenges to marine environments and human activity. This disrupts marine ecosystems by impeding the growth of other seaweeds and coral reefs, thereby reducing biodiversity. The fishing industry suffers from the entanglement of nets and obstruction of vessel navigation, leading to decreased catch volumes and economic losses. Tourism is negatively affected when seaweed washes ashore, spoils beach aesthetics, and emits unpleasant odors. Its decomposition depletes oxygen and disrupts the chemical balance of seawater, thereby stressing marine life. Aquaculture hampers the health and growth of farmed species, resulting in further economic losses. In addition, the removal and disposal of this seaweed incurs significant costs, burdening local economies. Continuous monitoring, effective management, dedicated policies, and research are crucial for mitigating these impacts [23,24,25].

Generally, seaweeds harvested in summer cannot be eaten or used as food, because the rise in sea temperature increases their cellulose content. Seaweed waste harvested in summer absorbs water molecules well through its cellulosic and amylose structures and increases the bonding strength with metal ions, making it suitable for manufacturing porous carbon structures that improve energy storage capacity [26]. To solve the increasing global seaweed waste, we will use practical and feasible technology to convert it into a high-value product to solve the problem of biomass pollution and manufacture it as an electrode material for high-performance supercapacitors.

Herein, we describe the fabrication of hierarchical graphitic carbon structures (HGCs) from seaweed waste. For the production of HGCs, nickel ions impregnated into seaweed (Sw/Ni^+^) were converted to a highly crystalline carbon structure with nickel nanoparticles through an annealing process. Numerous nano-sized pores and a large surface area (806 m^2^/g) were developed by etching the Ni particles, increasing the number of electrically active sites. HGCs also exhibited high capacitance when applied to supercapacitors and retained nearly all of their capacity after 10,000 cycles. Consequently, HGCs are high-performance electrochemical materials suitable for various energy applications.

## 2. Materials and Methods

### 2.1. Materials

Seaweed (Hizikia Fusiforme) was harvested from Wando, Republic of Korea. Nickel (II) chloride hexahydrate (98%) (NiCl_2_·6H_2_O), 1 N-hydrochloric acid, and ammonium peroxodisulfate were purchased from Sigma-Aldrich Co. (St. Louis, MO, USA), Ducksan Co. (Gwangju-si, Gyeonggi-do, Republic of Korea), and Kanto Chemical Co. (Tokyo, Japan), respectively.

### 2.2. Preparation of Nickel Ion-Impregnated Seaweed (Sw/Ni^+^)

The wet seaweed (Sw) was washed thoroughly with distilled water several times to remove any inorganic impurities and then ground into fine particles. The seaweed was then immersed in distilled water at ambient temperature for 3 days, and nickel salt was added at 100, 200, 300, and 500 phr (*phr: part per hundred parts of resin*) by weight. The resulting product was dried overnight at 40 °C in a vacuum oven.

### 2.3. Fabrication of HGCs

HGCs were prepared by the following steps. First, thermal annealing of the prepared Sw/Ni^+^ was performed at 1000 °C at a heating rate of 10 °C/min in a mixed gas (Ar/H_2_) flow and then placed in isothermal conditions for 30 min. After cooling to ambient temperature, the carbonized HGC/Ni (0) sample was immersed in a mild etchant to remove reduced nickel, and the solution was filtered and washed with distilled water several times. Finally, freestanding HGCs were obtained after drying at 40 °C in a vacuum oven for a day.

### 2.4. Characterization

The morphologies and microstructures of the samples were characterized using scanning electron microscopy (FE-SEM, Nano230, FEI, Hillsboro, OR, USA) at 15 kV, transmission electron microscopy (FE-TEM, Tecnai G2 F20 X-Twin, FEI) with an acceleration voltage of 200 kV, X-ray diffraction spectrometry (Normal XRD, D8 Advance, Bruker AXS, Billerica, MA, USA, with Cu Kα radiation), and laser scanning confocal micro-Raman spectrometry (AFM-Raman, alpha300s, WITec, Ulm, Germany, laser excitation at 532 nm). The specific surface area and pore volumes were determined using nitrogen adsorption–desorption isotherms (ASAP 2420 instrument, Micromeritics at 77 K, Norcross, GA, USA). The surface area and pore size distribution were calculated using the Brunauer–Emmett–Teller (BET) method, and the pore size distribution was also determined based on the density functional theory (DFT) model. X-ray photoelectron spectroscopy (XPS, K-alpha, Thermo Fisher, Waltham, MA, USA) was also performed in the energy range of 200 eV to 3 keV.

### 2.5. Electrochemical Characterization

Electrochemical measurements such as cyclic voltammetry (CV), galvanostatic charge–discharge, and cycle stability tests were performed using a conventional battery tester (WBCS 3000, Won-A-Tech, Seoul, Republic of Korea) under ambient conditions. For the electrochemical measurements, a three-electrode system consisting of a Pt mesh counter electrode, Ag/AgCl as the reference electrode, and HGCs on stainless steel as the working electrode (mass loading (5 mg on 1 cm × 1 cm stainless steel)) was used. All the electrochemical tests were performed in a saturated 1 M H_2_SO_4_ electrolyte. The working electrode was prepared by mixing 80 wt% HGCs, 10 wt% carbon black, and 10 wt% of polyvinylidenedifluoride (PVDF) in the presence of N-methyl pyrrolidinone (NMP). After thorough mixing, the paste was applied onto precleaned stainless steel and dried at 120 °C overnight in a vacuum oven. CV was performed at scan rates ranging from 2 to 100 mV/s. Galvanostatic charge–discharge measurements were performed at scan rates ranging from 0.1 to 5 A/g. The specific capacitances were calculated using the following equation:

$$C=\frac{1}{m\cdot \Delta V\cdot v}\int IdV$$

where C is the specific capacitance (F/g), *I* is the current (A), Δ*V* is the applied potential window (V), and *m* is the total mass of the active material (g).

## 3. Results and Discussion

**Figure 1:**
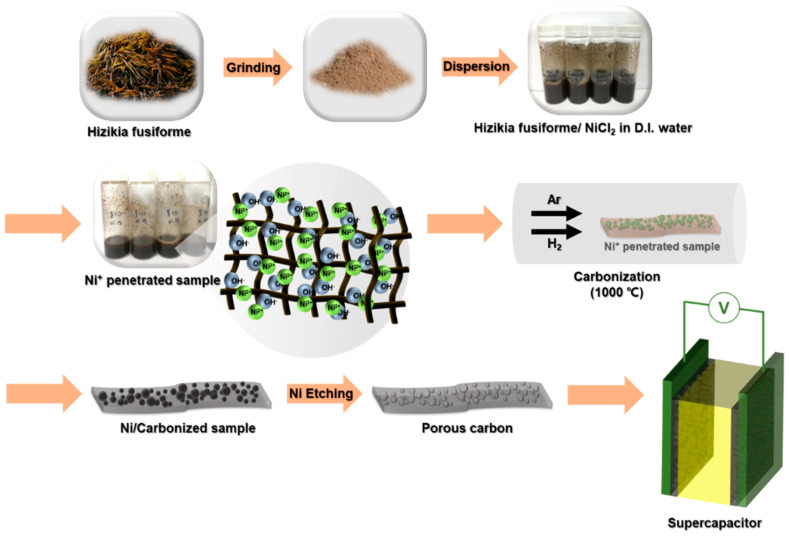
Schematic illustration for fabrication of HGCs from impregnated Sw/Ni^+^.

Figure 1 shows the fabrication process for the HGCs. First, they were washed several times with distilled water to remove impurities on the surface of the Sw and then finely ground. Subsequently, Sw was swollen in distilled water to improve its wettability. Sw, a marine plant, allows the easy diffusion of water and metal ions from the external environment into its interior. Furthermore, Sw is composed of numerous oxygen-containing chemical groups (such as –OH, –O–, =O, and –OOH), which are specific functional groups of major biological components such as proteins and amino acids. Most oxygen groups exhibit a negative charge owing to the presence of two lone pairs of electrons on the oxygen atom. Second, the swollen Sw was immersed in distilled water with nickel salt (NiCl_2_·6H_2_O) at ambient temperature for 3 days. Nickel cations were uniformly adhered to the oxygen groups inside and outside the swollen Sw through electrostatic attraction using water as a medium. The prepared mixture was slowly and perfectly dried overnight at 40 °C in a vacuum oven to prevent the oxidation of nickel ions and change in chemical structure. Finally, the nickel ion-impregnated seaweed (Sw/Ni^+^) was obtained.

Third, annealing the Sw/Ni^+^ complexes in a mixed gas (Ar/H_2_) at 1000 °C reduced the nickel ions (II) to nickel (0) and facilitated the formation of highly crystalline carbon using Sw as a carbon source on the surface of the nickel catalyst [27,28,29]. Finally, the nickel metal that grew on the carbonized Sw was removed using a mild etchant to obtain the HGCs. The process of producing highly crystalline porous carbon structures from Sw is simple and efficient, making it easy to use for industrial purposes.

**Figure 2:**
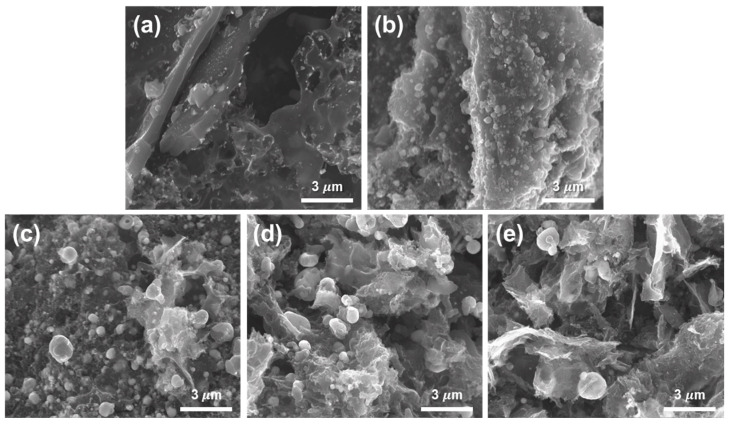
Morphological characterization of HGCs after annealing with different ratios of nickel salt: (**a**) 0, (**b**) 100, (**c**) 200, (**d**) 300, and (**e**) 500 phr, respectively.

Figure 2 shows a scanning electron microscopy (SEM) image of carbonized Sw/Ni^+^ with different nickel salt ratios. Among the samples obtained through the annealing process, the pristine sample with 0 phr Ni salt exhibited a relatively smooth surface with some structural features, and no significant particle formation was observed because of the absence of Ni, as shown in Figure 2a. With 100 phr of nickel salt, Figure 2b shows the initial formation of nickel particles on the surface and inside the sample. Small nickel particles began to appear, indicating that nickel converted from Sw to a carbon structure, acting as a metal catalyst. As the concentration of the nickel salt increased from 200 to 500 phr, there was a noticeable increase in the density and size of the nickel particles on the surface. Additionally, the overall morphology of the sample changed from an aggregated to a porous structure because of the increased conversion of Sw to carbon structures, as shown in Figure 2c–e. These results confirm that the formation of highly crystalline and high-quality porous carbon structures was improved by increasing the nickel salt concentration.

**Figure 3:**
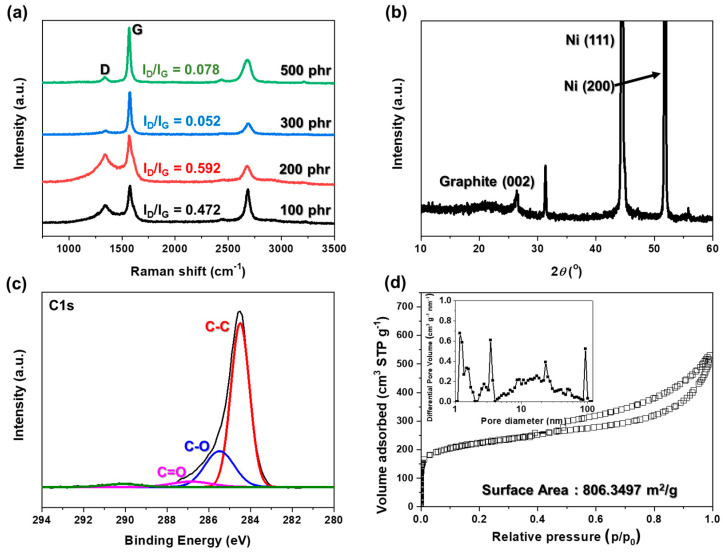
Characterization of HGCs. (**a**) Raman spectrum of HGCs with different nickel salt concentrations, (**b**) XRD patterns of HGCs at 300 phr nickel salt, (**c**) XPS spectra of C1s in HGCs at 300 phr nickel salt, (**d**) Brunauer–Emmett–Teller (BET) analysis of HGCs at 300 phr nickel salt.

Raman spectroscopy is a representative nondestructive analysis method primarily used to measure the quality of carbon. The Raman spectra shown in Figure 3a represent the state and quality of the HGCs. The characteristic bands of the carbon structure in the Raman analysis were broad D, G, and 2D bands at approximately 1350 and 1580 cm^−^^1^, respectively. The D band is associated with first-order zone-boundary phonons and is the disorder peak caused by defects in sp^2^ C–C bonds. The G band represents the main mode in graphene, indicating planar sp^2^ C–C bonds. With increasing nickel salt concentration, the intensity of the D band decreases up to 300 phr, followed by a slight increase at 500 phr. Concurrently, the intensity of the G band increases and narrows. The ratio of I_D_/I_G_ is widely used to determine the quality of the graphitic carbon structures. The minimum I_D_/I_G_ ratio is observed at 300 phr, which is the optimal composition ratio of seaweed to nickel salt for achieving high-quality carbon structures [30].

The XRD pattern of the HGCs at 300 phr demonstrates the complete conversion of the nickel ions implanted into the HGCs to nickel metal following annealing, as shown in Figure 3b. The reduction of nickel ions to nickel metal was confirmed by sharp and distinct peaks at (200) and (111), indicating the presence of nickel metal. In addition, the characteristic peak of graphite was observed at 26° (002). These results suggest that the nickel ions were successfully reduced to nickel metal and that Sw was converted to graphitic carbon [31,32].

X-ray photoelectron spectroscopy (XPS) was used to investigate the surface chemistry and electronic states of the carbon structures. This technique provides crucial information regarding the specific chemical groups present, such as oxygen-containing functional groups, and allows the detection of various types of structural defects, including vacancies and disordered regions, thereby offering a comprehensive understanding of the chemical composition and electronic properties. Figure 3c shows the XPS spectrum of C1s centered at 284.4 eV for carbon after the annealing process. A strong sharp peak (C–C) at 284.4 eV indicates high-quality graphite. Smaller peaks at 285.4 and 286.8 eV are due to C–O and C=O bonds from the cellulose and amylose in Sw. These findings suggest that the HGCs contain only carbon and oxygen without impurities. Additionally, the full width at half maximum (FWHM) of the C–C peak decreased to 1.03, indicating high crystallinity. Therefore, HGCs have a graphitic carbon structure suitable for use as electrode materials [33].

Figure 3d shows the surface area and pore size distribution of HGCs, analyzed using the BET analysis. The adsorption isotherms indicate the presence of both micropores and mesopores, as shown by the combination of a Type I isotherm, with a sharp rise at P/P_0_ ≤ 0.01, and a Type IV isotherm, which features a hysteresis loop from P/P_0_ = 0.5 to 1.0. The HGCs have a range of pore sizes and a high specific surface area of 806.35 m^2^/g, which supports effective electrolyte penetration and ion adsorption, improving electrode performance. The inset in Figure 3d shows the pore size distribution, indicating that the micropores, mesopores, and macropores were evenly distributed. This even distribution is important for fabricating electrode materials with excellent properties [34].

**Figure 4:**
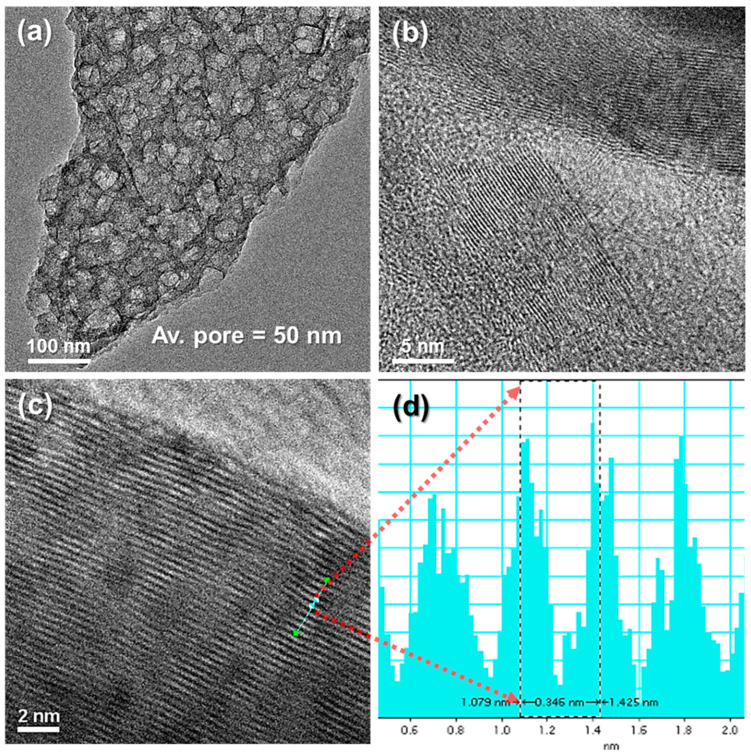
Transmission electron microscopy (TEM) image analysis. (**a**–**c**) TEM images of freestanding HGCs (300 phr) with different magnification and (**d**) d-spacing of carbon layer in HGCs.

Numerous spherical nickel particles were grown in the HGCs during annealing, but voids of various sizes were clearly visible in Figure 4a. The TEM image of the porous carbon used in this study shows the morphological changes after the removal of nickel metal immersed in a mild etchant. The first major feature of nanoporous carbon is that it facilitates electrolyte penetration through irregularly large pores throughout the carbon structure and provides a wider electrochemically active site. Second, the prepared porous carbon has a high surface-area-to-volume ratio, which contributes significantly to the adsorption–desorption process during electrochemical bilayer formation. A multi-graphitic layer was formed around the disordered pores of the HGCs, which exhibited good electrical conductivity, as shown in Figure 4b,c, and was also confirmed with a d-spacing of 0.34 nm in Figure 4d. The morphological analysis of the HGCs using TEM showed that they are very suitable as electrodes for supercapacitors because of their high crystallinity [35].

**Figure 5:**
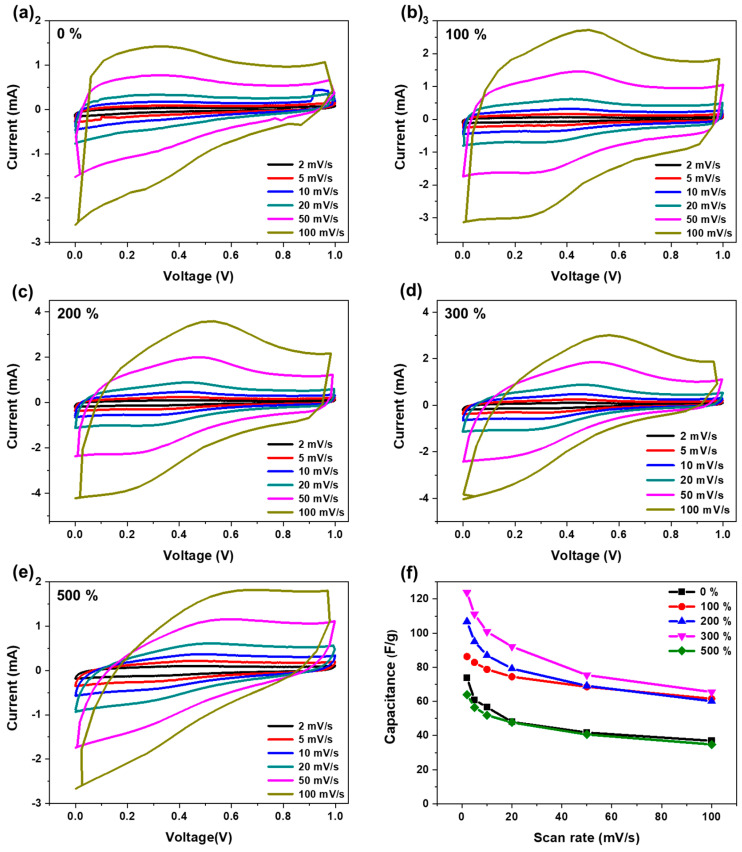
Electrochemical performance of HGC electrode. (**a**) CV curves of HGCs with different ratios of nickel salt: (**a**) 0, (**b**) 100, (**c**) 200, (**d**) 300, and (**e**) 500 phr; (**f**) variation in specific capacitance versus scan rate.

Figure 5a–e show the CV characteristics of the EDLC using the HGCs at a wide scan rate of 5–100 mV/s. At a relatively low scan rate, the CV curves exhibited a quasi-rectangular shape. This is because HGCs have pores of various sizes that facilitate the reversible adsorption–desorption reaction of the electrolyte and ions. However, at high scan speeds, the peaks were irregularly shaped. This is because, within a short time at a high scan speed, ion diffusion could not properly access the surface of the HGCs. Additionally, the deviation from the typical rectangular shape of the CV curves suggests the presence of pseudocapacitance, likely due to redox-active functional groups such as hydroxyl, carbonyl, or carboxyl on the surface of the HGCs. These faradaic reactions, which involve charge transfer across the electrode–electrolyte interface, enhance the capacitance. The resulting non-rectangular CV profile reflects the hybrid charge storage mechanism of HGC-based electrodes, combining both double-layer capacitance and pseudocapacitance [36,37]. Figure 5f shows the variation in the capacity according to the scan rate. Supercapacitors using HGCs as electrodes experience difficulties in the diffusion and pore diffusion of electrolyte ions into the electrode structure as the scan speed increases, inefficient interaction between the electrolyte and the HGCs occurs, and the non-electrolytic capacity decreases. At a nickel salt concentration of 300 phr, the HGCs exhibited maximum capacity and stability. This suggests that a significant number of mesopores formed within the HGCs at this concentration, facilitating a smooth and efficient ion transfer pathway. Consequently, the accessibility of the electrolyte to the microporous regions is significantly enhanced [34,38,39].

**Figure 6:**
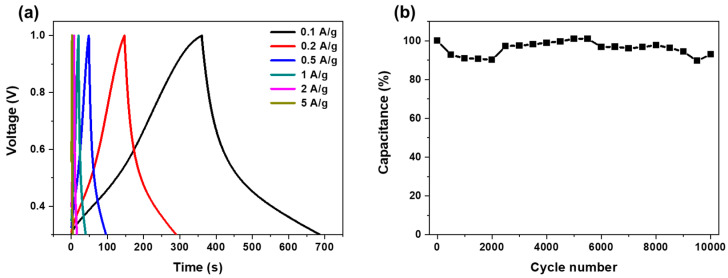
(**a**) Galvanostatic charge–discharge curve at different discharge current densities. (**b**) Capacitance retention versus cycle number at 2 A/g.

The electrochemical properties of the HGCs, which are relevant for their potential use as electrode materials for supercapacitors, were further examined. Figure 6a illustrates the galvanostatic charge—profiles of porous carbon at current densities ranging from 0.1 to 5 A/g. At lower current densities, the charge–discharge characteristics exhibited asymmetry owing to the differing rates of electrolyte adsorption and desorption. Notably, even at higher current densities, the shape and characteristics of the curve remained relatively consistent, indicating robust high-rate performance. This performance was attributed to the porous structure of the HGCs, which effectively enhanced the energy density of the electrode material.

Long-term cycling stability is a key requirement for electrode materials used in electrochemical applications. As shown in Figure 5b, the specific capacity at a current density of 2 A/g remained stable over 10,000 cycles. The HGC-based supercapacitor retained 92.4% of its original performance after 10,000 cycles, demonstrating remarkable life-cycle stability and highlighting the potential of HGC-based electrode materials for practical use in electrochemical capacitors [40,41,42].

## 4. Conclusions

In this study, HGCs were successfully fabricated from seaweed waste impregnated with nickel ions. The production process involves the conversion of nickel ions into a highly crystalline carbon structure via annealing, followed by the development of numerous nano-sized pores and a large surface area through nickel etching. The resulting HGCs exhibited exceptional electrochemical properties, including high capacitance, a large specific surface area (806 m^2^/g), and robust cycling stability, retaining 92.4% of their capacity after 10,000 cycles. These characteristics highlight the potential of HGCs as high-performance electrode materials for supercapacitors and other energy storage applications. Moreover, utilizing seaweed waste not only addresses the environmental challenges associated with marine biomass but also provides a sustainable pathway for producing efficient energy storage materials. This innovative approach aligns with the broader goals of sustainability and resource optimization in the field of renewable energy technologies.

## Data Availability

The data presented in this study are available in this article.
